# Does Tobacco Impair Sight?

**Published:** 1885-09

**Authors:** W. Franklin Coleman

**Affiliations:** M. D., M. R. C. S., England. 70 Monroe Street; Chicago


DOES TOBACCO IMPAIR SIGHT? *A Paper Read Before the Chicago Medical Society, August 3rd, 1885. By* W. FRANKLIN COLEMAN, M. D., M. R. C. S., *England. Chicago.*

*Mr. President and Gentlemen:* Your secretary has suggested that every candidate for, or newly-elected member of, this society should be obliged to read a paper as an initiation. Should you also think it a fit and proper obligation, a sufficient excuse for this youthful conduct is not wanting.

To consider the physical effects of a poisonous weed, food for neither man nor beast, yet consumed alike in every quarter of the globe, by savage and sage, saint and sinner, is a theme of no little consequence.

Tobacco belongs to the family solanaceæ, which claims such relations as hyoscyamus, belladonna, stramonium, and, curiously enough, the potato. Its important active principles are an alkaloid called nicotine, a volatile oil, nicotianin.

Von Brock says: "It is from smoking tobacco that nicotine poisoning chiefly arises—the smoke itself containing the nicotine. A great deal of it accumulates on the lower part of the pipe, and the remains of cigars are much more impregnated with it than parts fresh-smoked."

The observations of Claude Bernard, that nicotine at first produces contraction of the arteries, and later on the vessels become distended, agree with the views of Uspensky, who, from physiological researches, concludes that nicotine first stimulates then paralyzes the vasomotor centres.

From personal experience, and the literature at my command, I know of no more constant detrimental effect of the use of tobacco than more or less impairment of vision—so-called amblyopia.

The use of tobacco is so frequently associated with drinking to excess that it is questioned by some whether tobacco alone ever produces defective eyesight; whether the alcohol is not the chief or only etiological factor. In England there is a general belief among surgeons that tobacco is the more frequent cause of the amblyopia, while in America alcohol is more generally thought to be the principal or only agent.

Dr. Webster, of New York, in an able paper reporting twenty cases of amblyopia from the abuse of alcohol and tobacco, remarks: "That the abuse of alcohol and tobacco alone, or of alcohol and tobacco combined, may produce impairment of vision, no physician acquainted with the subject will, I think, venture to deny. Some, however, doubt that tobacco alone ever causes impairment of vision, and indeed it is difficult to demonstrate that it ever does."

Dr. Webster apparently holds the above views so firmly that he quite overlooks the fact that two of his cases prove that tobacco alone does produce amblyopia, while there is not a single case to show that alcohol alone was the cause of impaired sight—for all who used alcohol also smoked to excess. Take, for example, case 7, C. McK., æt. 49; has smoked ten to fifteen strong cigars daily for ten years; *occasionally* drinks a glass or two of gin; V=1-16 each eye. Incipient atrophy of optic nerve. Case 12, æt. 60; sight failing over a year; has smoked a strong pipe most of his waking hours for more than forty years; has rarely tasted liquor; V=1-40 each eye; brick dust atrophy of both optic nerves; ordered to stop tobacco and return in a week; the vision in right eye doubled; in left all but doubled.

Observers unanimously agree that amblyopia is produced only by the *excessive*, not the *occasional*, use of alcohol or tobacco, or tobacco and alcohol combined. Hence, in the case of the "occasional drink," and in 12, the "rare taste" of liquor cannot be held responsible for the loss of sight.

So many competent observers believe in alcohol-amblyopia, I readily accept their view, but in my experience the opportunity for making out a case against alcohol is rare, as the tippler is almost invariably a smoker. The case against tobacco alone can be much more frequently proven, since it is not uncommon for an inveterate smoker to be a teetotaller, or to taste liquors but rarely.

The celebrated philanthropist, McKenzie, who was first to bring to public notice the effects of tobacco upon vision, wrote in his text-book of 1840: "I have already had occasion to repeatedly hint my suspicions that one of the narcotic acrids which custom has foolishly introduced into common use, namely, tobacco, is a frequent cause of amaurosis." More recently, such men as Wordsworth, Hart, Hutchinson, Forster, Hirschberg, Sichel, Luriero, Jackson, and a host of others, have given much attention to the effects of tobacco, and they maintain that it gives rise to impaired sight and even blindness.

Mr. Hutchinson, ever cautious, would not express an opinion in 1870, but remarked to me at Moorfields, in 1876, that he had come to think the effect of alcohol antagonistic to tobacco as a cause of amblyopia, unless the alcohol is taken in such excess as to produce degenerative effects of the system. He had seen amblyopia more frequently and more advanced in smokers who abstained from than in those who used alcohol. Dr. Berry, of Edinburg, holds a similar opinion.

In tobacco-amblyopia, the patient, usually a man past thirty, complains of gradual failure of vision; the defect, unless very considerable, being much the same in both eyes. The sight is generally described by the patient and surgeon as better in a dull light. Dr. Berry, after careful testing, concludes this is a mistake, having found the acuity of vision really less when the light is diminished. Central amblyopia is a very characteristic symptom, direct vision alone, as a rule, being impaired, while the indirect is only slightly or not at all affected; hence, the person is enabled to get about without the least difficulty. Förster first drew attention to red-blindness, in the center of the field of vision, as a pathognomonic symptom. In the less severe cases, this central color scotoma may be recognized when a central blind-spot for white light or form cannot be detected. The color-blindness extends to the periphery of the field only in the more serious cases. The impairment of sight may occur with or without other symptoms of tobacco-poisoning of the brain, and so forth, such as loss of memory, headache, sleeplessness, loss of appetite, palpitation, etc.

The ophthalmoscope in the majority of cases, it is said, cannot detect any pathological change. When abnormal appearances are seen they are wholly confined to the optic disc. At first it may look smoky or hazy. This stage of hyperæmia is followed by decoloration of the temporal half of the papilla, which later extends to the whole disc and occasionally terminates in complete atrophy and blindness. Once I have seen the very rare condition, perivasculitis According to Hutchinson, the stages of the disease of the nerves occupy from four to twelve months.

In 1867 Mr. Hutchinson, reporting thirty-seven cases of primary white atrophy of the optic nerve, all that had come under his care during three years, attributed twenty-seven to the effect of tobacco. Dr. Green, of St. Louis, says a very large proportion of the cases of optic nerve atrophy, which have fallen under his observation, have been in cigar makers and workmen in the tobacco factories, who habitually use tobacco very freely by smoking and chewing.

Amblyopia is more readily produced by the strong tobaccos, such as the "Schag" of England. Chewing is more dangerous than smoking (Nettleship). Berry is disposed to think that the blindness very rarely occurs if smoking be only indulged in after a meal.

In functional cases, vision will be completely restored if tobacco is given up, and alcohol, should it be used in excess. If there be marked ophthalmoscopic changes in the nerve, the defect of vision may remain stationary, but, in my experience, vision has nearly always improved under treatment. Blindness very rarely results, perhaps only in case of progressive atrophy of the nerve.

Little is evidently known in regard to the pathology of tobacco amblyopia. The view of Leber, that there is an anomaly in the blood-supply to the superficial layer only of the optic nerve, seems, at the same time, to account best for the *central scotoma* and the generally favorable course of the disease; for, it will be remembered, the external optic nerve fibers are distributed to the center of the retina, while the inner fibres of the nerve supply the eccentric portions of the retina. Berry well maintains that the disease is essentially functional that even interstitial change in the nerve is not the direct cause of the amblyopia, in proof of which he cites the well-known fact that pallor of the disc remains even when vision recovers. The change in the nerve and the amblyopia, he attributes at once to vasomotor disturbance or other functional disorder.

In regard to treatment, the withholding of tobacco is an evident requisite. Opinion is divided as to the benefit of the hypodermic use of strychnia usually resorted to. If the strychnia is pushed to the point of toleration or spasm, usually requiring 1-10 grain three times a day, often 1-8 grain, occasionly 1-4 grain, I believe recovery is always hastened and often occasioned. The success of galvanic electricity claims more attention as a remedy. Dr. Bradford, of Boston, related to me a case of atrophy of the optic nerve, from tobacco, in which the blind eye recovered useful vision by the use of chloride of barium, after strychnia, electricity, and all the usual remedies had failed.

Since I have more particularly recorded cases, I find in my note books, among 1,824 eye-patients, forty-six who had partial to total loss of sight, accompanied so far by conditions similar to those noticed in tobacco-amblyopia, as to present either no ophthalmoscopic changes in the eye, or else hyperæmia, decoloration or atrophy of the optic papilla.

These forty-six cases may be thus classified :

Males, 33 ; females, 13.

Cases referred to tobacco alone, 13.

" " " " and alcohol, 9.

" " " other causes, 24.

All the tobacco, and tobacco and alcohol cases, occured in males.

Of those referred to other causes, ten were males, fourteen females.

Cases in which there was hyperæmia, discoloration or atrophy of the disc :

Tobacco, males, 12 ; females, 0.

Alcohol and tobacco, males, 9 ; females, 0.

Other causes, males, 7 ; females, 11.

Cases which presented no ophthalmoscopic changes :

Tobacco, males, 1 ; females, 0.

Tobacco and alcohol, males, 0 ; females, 0.

Other causes, males, 3 ; females, 3.

In regard to the above, the notable absence of any case of pure alcohol amblyopia may be accounted for by the alcoholic patient being, invariably, a heavy smoker.

I must admit, females were not questioned as to the alcohol or smoking habit, there being no reason to suspect them of such male virtues. Again, my experience of so large a proportion of patients as twenty out of twenty-one, who presented changes in the optic disc in contrast to the rule that no pathological changes are seen in tobacco-amblyopia, may be due to the advanced stage of the disease when first examined. This experience agrees, however, with that of Dr. Webster, who reports marked ophthalmoscopic changes in all of his twenty cases.

In illustration of tobacco-amblyopia I will refer briefly to four patients who were not addicted to drinking—and to one other.

J. T., age 44. Consulted me in February, '83, on account of defective sight, of eight months' duration. Vision, right eye 1/3, left eye 1/2. In reading, the right eye sees only the first part of a word, and the left only the latter part—temporal hemiopia.

Has smoked six pipefuls daily for the past fifteen years; health good. The temporal half of each optic disc is pale.

Advised to stop smoking, and strychnia prescribed.

Two months later, he remarked he had followed advice and the sight was quite restored.

CASE 2. H. O., age, 21. Came under my care December 28, '83. His sight has gradually failed during the past fifteen months, and at present he can barely distinguish light. No symptoms of brain or spinal disease. No history of syphilis or other poisoning, except tobacco. Health very good. Has for four years past smoked six to seven pipefuls of tobacco daily, and chewed one-fifth of a pound weekly.

There is advanced white atrophy of both optic papillæ.

Ordered 4 minims of a four-fifths per cent. solution of strychnia to be given hypodermically twice a day, and increased one minim daily—and tobacco to be discontinued.

Five weeks later: The strychnia has been increased to 1-5 of a grain, producing only occasional muscular spasm. As vision has not improved, galvanic electricity directed to be applied daily to the closed lids, temples and nape of the neck.

Repeat strychnia 1-12 of a grain, three times a day, by the stomach and increase the dose.

Three weeks subsequently, Feb. 27th, he is taking 1-5 of a grain of strychnia three times a day and having electricity applied.

The vision is now increased to 1-70 in each eye, and the patient is able to see his way in the street very well.

April 7th. Discharged with sight remaining the same. It is now reported that a good deal of smoking was slyly indulged in.

CASE 3. Nov. 25, '80. J. McKay, aged 31. Complains of failing sight for the past three months, which is now reduced to 1-17 in each eye. He began to smoke at eleven years of age, and for three years past has averaged ten pipefuls a day. Very rarely tastes liquor. Ophthalmoscopic examination.

Both optic discs are hyperæmic. Advised to give up tobacco, have temples cupped, and take K. I.

The patient returned in six weeks.

Has stopped smoking and gained twelve pounds in weight. Vision has improved from 1-17 to 1-5 in each eye.

The temporal side of each disc is now pale.

Jan. 25th. There now present white lines (of perivasculitis) at the margins of retinal vessels on and near the discs. Strychnia prescribed.

CASE 4. W. S., aged 24. Consulted me in October '79. Noticed nine months ago in school he could not read with the right eye; three months after observed the same defect in the left, but two weeks later could see to read fairly well. A week later still, the eyes again failed. Is anæmic and nervous, but considers his health pretty good. Has smoked six to eight pipefuls daily from the age of fifteen until two years ago, and since then three to four pipes a day.

Right eye can count fingers within six inches only on the temporal side. Left eye vision=20-140.

Externally and internally the eyes appear normal.

*Treatment.*—Strychnia hypodermically twice a day. Patient discharged nine days with vision of right eye improved eight times and doubled in the left.

Allow me to cite a case which seems to confirm the view that alcohol counteracts the effect of tobacco:

C. M., aged thirty-one, relates, July, 12, '84, that his sight has been failing for six weeks. That he has smoked six to eight pipes a day for the past seventeen years, and has been a hard drinker for eight years, until three months ago, since which time he has abstained completely. The patient *volunteered* the remark, "I think stopping spirits suddenly caused my sight to fail." Vision had always been good up to six weeks ago (*i. e.*, six weeks after giving up spirits).

*Treatment*—Take strychnia, gr. 1-20, three times daily, and advised to stop smoking in preference to returning to spirits.

In six days vision doubled in both eyes. Take strychnia, gr., 1-16 *ter die.*

October 9th.—Vision of right eye has increased in three months from 1-10 to ½, and in left from 1-10 to ⅔.

Let us return to the question—Does tobacco impair sight?

Among the authors of text-books in my possession, who express their belief in tobacco amblyopia, are Scotch, American, German and French, viz: McKenzie, Wolfe; Gowers, Wells, Nettleship; Noyes, Williams; Stellwag, Schweigger, Greürfeld, Mittendorf; Mayer, De Wecker. The only two authors I know who dissent from the general view, are Carter and Lawson (English).

Mr. Carter quotes a letter from Dr. Dickson, of Constantinople, to the effect that the consumption of tobacco in that city averages three pounds weight per head, monthly, but amblyopia is a rare affection.

Dr. Hubsch, oculist, in Constantinople, also writes him, "I have never attributed amaurosis to the abuse of tobacco."

Mr. Carter adds, "I have obtained the same kind of negative evidence from Egypt and India, and in the face of it I do not attach much importance to the fact that several patients who have suffered from severe atrophy have been great smokers." The last statement is a little weakened when he adds, "If a patient who consults me on account of severe atrophy is a smoker, I always advise him to lay aside tobacco."

It may be maintained by some that impaired sight, the use of tobacco, and the wearing of leather boots, for example, are coincidences, "only that and nothing more."

But when we find, in common with so large a number of competent observers, that impaired vision, accompanied or not by apparent changes in the optic nerve, so frequently occurs without any cause to which it can be attributed, except the use of tobacco, and that the sight so frequently improves when it is given up, without other treatment, or any change in the habits or surroundings of the patient, we can scarcely hope for stronger evidence of the cause of the amblyopia.

Again, observers agree that a large class of cases presenting central color blindness, and a diminution of direct visual acuity, have a toxic origin, such as tobacco, alcohol, etc., and that these symptoms are most frequently produced by tobacco. Berry says he has looked out for the symptoms of tobacco amblyopia in women for the past five or six years and has only met with them in three cases. These three women smoked to excess but did not drink. Now, it is well known that women in Great

Britain seldom smoke, but lamentably often feel disposed to take spirits. They thus afford, negatively, evidence for tobacco-amblyopia, and proof that the above symptoms are less frequently produced by alcohol than tobacco.

Further, the abundant testimony that tobacco acts detrimentally upon other parts of the nervous system, even causes myelitis and atrophy of the cord, affords strong circumstantial evidence for attributing neuritis and atrophy of the optic nerve likewise to tobacco.

Bean describes eight cases of angina pectoris in which the attacks ceased when smoking was stopped, and returned when the patient began to smoke.

Most of you are doubtless familiar with the "tobacco heart" of Dr. Bowditch.

Erb, of Heidelberg, says: "Various authors adduce excessive tobacco smoking among the causes of tabes dorsalis."

Those who disbelieve in tobacco-amblyopia think their view is strongly supported, insomuch as among the army of smokers there are relatively a few only whose sight becomes affected. The absence of amblyopia in Constantinople, where smoking is so general, seems to support more strongly still the above view. But the *mild quality* of the tobacco and the *mode* of smoking in Turkey differ so much from the "shag" of England, and the method of smoking in England and America, as possibly to account for the immunity of the Turk.

It has been experimentally determined by a French pharmacist, Malapert, that a dry cigar yields, while burning, a very small amount of watery vapor, the smoke cools rapidly and allows the condensation of nicotine before it reaches the mouth. Hence the first half of a cigar or pipe smokes more mildly than the second, in which a certain amount of watery vapor and nicotine freed by the first half is deposited.

From this it is evident the cigar smoker may, if he "throw away the worser part of it, live the purer with the other half."

Sir Henry Thompson tells us "the ladies of Constantinople smoke fifty cigarettes a day, merely taking a few whiffs from each, then throw the cigarette away, and considers little harm ensues from such smoking."

In the case of the pipe, the quantity of nicotine that will reach the mouth may possibly be reduced to the safety-point by smoking through long stems or water.

If the Turk, who ornamented the geography of our early school days, still survives, the long, flexible stem of his magnificent pipe may at this moment be the safeguard of his optic nerves.

The custom among the Turks of drawing the smoke through water in the nargilleh or water-pipe, a process called "bubble, bubble," is a very valid additional reason for their freedom from tobacco-amblyopia.

Peculiarity of race, idiosyncrasy of constitution, nervous exhaustion, impaired nutrition and other debilitating causes may or may not account for the elective affinity of tobacco, alcohol, lead, silver, mercury, nitro-benzol, sulphide of carbon, quinine, salicylic and osmic acids, for the eyes of certain individuals. But to deny that these substances produce amblyopia because so large a number are exposed with impunity, is as reasonable as to deny small-pox can reproduce itself because the majority of people escape, or that cirrhosis of the liver is caused by alcohol because so many dram drinkers maintain sound livers, or that cold and wet can produce rheumatism because so few of the exposed multitude suffer.

If this paper should be the means of securing your views, and afford any hints concerning the entity and nature of tobacco-amblyopia, I shall be fully repaid and feel that your time has not been squandered.

70 Monroe Street.

